# Post-traumatic stress disorder and risk for hospitalization and death following COVID-19 infection

**DOI:** 10.1038/s41398-022-02156-w

**Published:** 2022-11-22

**Authors:** Kristen Nishimi, Thomas C. Neylan, Daniel Bertenthal, Emily A. Dolsen, Karen H. Seal, Aoife O’Donovan

**Affiliations:** 1 Mental Health Service, San Francisco Veterans Affairs Health Care System, 4150 Clement Street, San Francisco, CA 94121 USA; 2 Department of Psychiatry and Behavioral Sciences, University of California San Francisco, 675 18th St., San Francisco, CA 94107 USA; 3 Integrative Health Service, San Francisco Veterans Affairs Health Care System, 4150 Clement Street, San Francisco, CA 94121 USA; 4 Departments of Medicine and Psychiatry, University of California San Francisco, 533 Parnassus Ave, San Francisco, CA 94143 USA

**Keywords:** Depression, Bipolar disorder, Schizophrenia

## Abstract

Post-traumatic stress disorder (PTSD) is associated with an increased risk for physical illnesses and early mortality. However, we do not know if it also increases the risk for adverse outcomes of coronavirus disease 2019 (COVID-19). In this retrospective cohort study, we examined associations of PTSD and other psychiatric disorders with risk for hospitalization and death in the 60 days following a COVID-19 infection in 228,367 U.S. Department of Veteran Affairs (VA) patients who tested positive for COVID-19 between February 2020 and August 2021 (age m = 60.6, 89.5% male). Generalized linear models estimated associations of PTSD and other psychiatric disorders with outcomes following a positive SARS-CoV-2 test, adjusting for socio-demographic, medical, and behavioral factors. Among 228,367 VA patients, 25.6% had PTSD, and 28.2% had a psychiatric disorder other than PTSD. In the 60 days following a positive COVID-19 test, 15% of patients were hospitalized, and 6% died. Patients with PTSD had an increased risk for both hospitalization (adjusted relative risk, ARR = 1.18, 95% CI 1.15–1.21) and death (ARR = 1.13, 95% CI 1.08–1.19) relative to those with no psychiatric disorders, adjusting for socio-demographics. Estimates remained significant when models were additionally adjusted for medical comorbidities and smoking. Patients with other psychiatric disorders also had an increased risk of adverse COVID-19 outcomes, with larger effect sizes than PTSD in older (≥65 years) but not younger patients. In this large-scale study of VA patients, individuals with PTSD, and other psychiatric disorders, had heightened vulnerability to severe adverse outcomes of COVID-19; thus, individuals with PTSD should also be considered at higher risk for severe COVID-19 outcomes, and potentially prioritized for vaccination, screening, and early treatment intervention for COVID-19.

## Introduction

The coronavirus disease 2019 (COVID-19) pandemic has had an extraordinary impact on population health worldwide, contributing to more than six million deaths and many more hospitalizations since its inception [1]. Psychiatric disorders may be an important risk factor for worse COVID-19 outcomes, like hospitalizations and deaths, due to medical comorbidities, poor health behaviors, and dysregulation in immune functioning [2, 3]. Indeed, emerging large-scale studies indicate a heightened risk for adverse outcomes of COVID-19 among those with some psychiatric disorders [4–6]. Summarizing these studies, meta-analyses have confirmed that psychiatric disorders are associated with higher COVID-19 severity as indexed by hospitalizations and death [4–6], with meta-analytic adjusted odds ratios of 1.75 (95% CI 1.20–2.54) for hospitalization and 1.26 (95% CI 1.12–1.42) [5] to 1.38 (95% CI 1.15–1.65) for death [4]. Despite the increased risk for traumatic stressors during the pandemic and the strong potential for post-traumatic stress disorder (PTSD) to worsen disease outcomes in COVID-19 [7–9], we do not know if patients with PTSD should be considered a vulnerable group.

Multiple lines of evidence indicate that PTSD specifically could increase the risk for adverse outcomes of COVID-19. First, PTSD is associated with deleterious health behaviors (e.g., smoking, excess alcohol use, physical inactivity, and poor diet) [10], which could adversely impact COVID-19 outcomes. Second, PTSD is associated with elevated chronic peripheral inflammation [9, 11], reduced anti-inflammatory glucocorticoid signaling to immune cells [12], and accelerated cellular aging as indexed by telomere length [11, 13]. Overall, these features of PTSD could increase the risk for negative COVID-19 disease sequelae, including more severe disease and COVID-19-related cytokine storm syndrome [14, 15]. Third, PTSD may be associated with increased somatization or anxiety related to COVID-19 symptoms, which could increase the likelihood of hospitalizations for COVID-19 [16]. Finally, PTSD is associated with known medical risk factors for COVID-19 morbidity and mortality, including cardiovascular disease, hypertension, diabetes, and obesity, via similar physiological and behavioral mechanisms [7, 8, 17, 18]. Importantly, PTSD may even increase the risk for chronic medical conditions to a greater extent than other psychiatric disorders [7]. Although other psychiatric conditions have also been linked to physical comorbidities, inflammation, and health risk behaviors [19], PTSD, in particular, is characterized by lower levels of circulating cortisol, which is a stress hormone with anti-inflammatory properties that may be beneficial in reducing the inflammatory activity that underlies many acute adverse outcomes of COVID-19 [12, 20]. Notably, one study early in the pandemic found that VA patients with PTSD were more likely to be tested, but less likely to test positive, for COVID-19 [21], relative to those without PTSD. However, we do not yet know if PTSD increases the risk for poor outcomes following a positive severe acute respiratory syndrome coronavirus 2 (SARS-CoV-2) test.

In the current study, we examined associations between PTSD and negative COVID-19 outcomes, as indexed by risk for 60-day hospitalizations and deaths following a positive SARS-CoV-2 test (i.e., COVID-19 infection) in the U.S. Department of Veteran Affairs (VA) healthcare system. Military veterans overall represent a vulnerable group at high risk for COVID-19 complications resulting from socio-demographic risk factors (e.g., age, sex, racial/ethnic composition) and relatively high rates of medical and psychiatric comorbidities [22, 23]. We hypothesized that VA patients with PTSD would have a higher risk for hospitalization and death following COVID-19 infection, compared to VA patients without psychiatric disorders and those with psychiatric disorders other than PTSD. To examine if PTSD was associated with COVID-19 outcomes independent of other known risk factors, we adjusted for a range of medical and health-related factors.

## Materials and methods

### Study design and participants

Data for this retrospective cohort study came from the US VA Corporate Data Warehouse (CDW), a regional database of patient data from across all VA healthcare facilities, and the VA COVID-19 Shared Data Resource (HSR RES 13–457), which contains information on all VA patients who had a SARS-CoV-2 test recorded in VA clinical notes or lab records. The COVID-19 Shared Data Resource has enabled rapid research across a range of COVID-19-related topics (e.g., refs. [24, 25]). Our analysis included all individuals who accessed the VA healthcare system for services between February 20, 2020 and August 17, 2021 with a positive polymerase chain reaction (PCR) SARS-CoV-2 test from the VA or from outside the VA, which was recorded in VA clinical notes (if multiple positive tests, the first positive test date was used), with at least 60 days of follow-up after the positive test; inclusion and exclusion criteria were established a priori. Of the 1,423,988 individuals who accessed VA care in the study period, 257,108 (18.1%) had at least one positive SARS-CoV-2 PCR test. Among these 257,108 patients, we excluded those who were not active in the VA in the 12 months before their positive test date to reduce missing diagnoses (*n* = 8872 excluded), those with less than 60-day follow-up after their positive test date to avoid false negatives on our outcomes (*n* = 16,661 excluded), those whose hospital admission dates were greater than five days prior to their positive test date to avoid outcomes related to other diagnoses (*n* = 431 excluded), and those missing information on the key covariate of body mass index (BMI) (*n* = 2777 excluded), resulting in an analytic sample of 228,367 (see Fig. S1 for sample derivation). This study was approved by the Committee on Human Research, University of California, San Francisco, and the San Francisco VA Health Care System Human Research Protection Program, and a waiver of informed consent was approved for the analysis of health records data.

### Measures

Independent variables included psychiatric disorders, defined as any clinical diagnosis of PTSD, depression, anxiety, adjustment disorder, alcohol use disorder, substance use disorder, bipolar disorder, psychotic disorder, attention-deficit hyperactivity disorder (ADHD), dissociative disorder, and eating disorder within the 5 years prior to the initial positive SARS-CoV-2 test. Diagnoses were identified from inpatient or outpatient clinical data based on International Classification of Diseases, Ninth Revision, Clinical Modification (ICD-9-CM), or ICD-10-CM codes (Table S1), coded on at least two separate occasions to minimize misclassification. Consistent with prior work, patients were classified into three groups [7]: (1) patients with PTSD alone or in combination with other psychiatric disorders; (2) patients with psychiatric disorders other than PTSD; and (3) patients with no psychiatric disorders.

Outcomes included hospitalizations and deaths recorded in the 60 days following COVID-19 infection. Hospitalization was defined as any acute inpatient hospital admission with patient isolation within the 60 days following a positive SARS-CoV-2 test (excluding admissions to nursing homes, community living centers, or other long-term care facilities, and psychiatric or spinal cord injury admissions). Death was defined as any death within the 60 days following a positive SARS-CoV-2 test.

Covariates were socio-demographic factors including age, sex (female, male), race (Black or African American, white, other race [Asian, American Indian or Alaska Native, Native Hawaiian or Other Pacific Islander] or unknown), and Hispanic ethnicity (Hispanic or Latino, Not Hispanic, or Latino, unknown). Medical comorbidities included obesity (BMI ≥ 35 closest in time to the positive test date); any record of the following medical conditions in the 2 years prior to the positive test date: diabetes, cardiovascular disease including hypertension, obstructive sleep apnea, chronic obstructive pulmonary disease (COPD), cancer, chronic kidney disease, liver disease, human immunodeficiency virus (HIV); and smoking (never smoker, current, or former smoker) based on ICD-9-CM or ICD-10-CM codes.

### Statistical analysis

We first examined distributions of covariates by psychiatric disorder diagnosis using *t*-tests and Χ^2^ tests. We then used multivariable generalized linear models (specifying Poisson distribution and log link and robust error variance) [26] to determine whether PTSD or other psychiatric disorders were associated with the relative risk (RR) of 60-day (1) hospitalization and (2) death following a positive SARS-CoV-2 test. The reference group for all models was those with no psychiatric disorders, although we additionally examined differences in the effect sizes for PTSD compared to other psychiatric disorders. As age may not be linearly associated with risk for COVID-19 outcomes [27], we examined both linear and quadratic effects of age and found that including both age and age squared resulted in the best fitting model. Thus, models first adjusted for socio-demographics that are potential confounders (i.e., age, age squared, sex, race, and ethnicity) (Model 1), and then additionally adjusted for factors that may be confounders or pathway variables (i.e., medical comorbidities, smoking) (Model 2).

Older individuals are more likely to be hospitalized or die following COVID-19 infection [27], thus, we ran secondary analyses with the sample stratified at age 65. Additional secondary analyses examined the association between any psychiatric disorders (i.e., diagnosis of any of the included psychiatric disorders including PTSD) and risk for both outcomes relative to no psychiatric disorders, and between each individual disorder and risk for both outcomes relative to no psychiatric disorders. Although we included ADHD, dissociative disorder, and eating disorder diagnoses to capture a wide range of psychiatric disorders, we did not estimate individual effect estimates for these disorders given their low prevalence in the sample (ADHD prevalence = 2.3%, dissociative disorder prevalence = 0.4%, eating disorder prevalence = 0.3%) leading to a risk of imprecise estimates. We additionally ran individual psychiatric disorder models stratified at age 65 to determine if there were differences between effect estimates observed for older versus younger individuals. To examine age differences further, we also stratified the sample at <40 years and 40–64 years and reran the primary analyses. Given the overlapping symptoms and high cooccurrence of diagnoses of PTSD and adjustment disorder (44.7% of those with adjustment disorder also had PTSD, and 22.3% of those with PTSD also had adjustment disorder), we performed a sensitivity analysis examining associations of PTSD or adjustment disorder with risk for COVID-19 outcomes. As COVID-19 vaccine rollout began during follow-up, we performed sensitivity analyses that excluded individuals with breakthrough infections, conservatively classified as any positive SARS-CoV-2 tests among patients >14 days after receiving one dose of any COVID-19 vaccine (*n* = 7918 excluded). Data were prepared with SAS 9.4, and analyses were conducted with Stata 15.1; all tests were two-sided, and an a priori threshold of *p* < .05 was considered for statistical significance. The code used to generate results in this study are available from the corresponding authors upon reasonable request.

## Results

The analytic sample of 228,367 patients with positive SARS-CoV-2 tests was 61 years old on average, 89.5% male, 67.4% white, 22.4% Black or African American, 10.2% other or unknown race, and 9.8% Hispanic or Latinx (Table 1). Regarding medical and behavioral risk factors for COVID-19, 72.1% of the sample had one or more medical comorbidities, 22.8% were obese (BMI ≥ 35), and 61.1% were current or former smokers. Just over one quarter (25.6%) of the sample had a PTSD diagnosis in the 5 years preceding their positive SARS-CoV-2 test, and an additional 28.2% had psychiatric diagnoses other than PTSD. There were high levels of psychiatric comorbidity, with 82.2% of those with PTSD having at least one other psychiatric diagnosis. Among those with either substance or alcohol use disorder, 84.8% had another psychiatric diagnosis. Psychiatric disorders varied by age: those with PTSD were younger on average (mean age 55) than those with other psychiatric disorders (mean age 58) and those without psychiatric disorders (mean age 65). Consequently, the prevalence of some medical comorbidities across the psychiatric disorder groups was likely confounded by age (e.g., older patients were less likely to have psychiatric disorders but also more likely to have cardiovascular disease, despite PTSD and psychiatric disorders being associated with cardiovascular disease risk in age-stratified groups) [10]. In the 60 days following the positive COVID-19 test, 15.0% of the sample was hospitalized, and 6.0% died.

**Table 1:** Distribution of socio-demographic factors and medical comorbidities among 228,367 VA patients.

	Full sample	No psychiatric disorders	Other psychiatric disorders	PTSD	
	*n* = 228,367	*n* = 105,405 (46.2)	*n* = 64,395 (28.2)	*n* = 58,567 (25.6)	
Covariate	No. (%)	No. (%)	No. (%)	No. (%)	*p*-value
Age, *m (SD)*, in years	60.6 (16.3)	65.2 (15.4)	58.4 (16.1)	54.7 (15.7)	<0.001
Sex					
Female	23,901 (10.5)	6854 (6.5)	8739 (13.6)	8308 (14.2)	<0.001
Male	204,466 (89.5)	98,551 (93.5)	55,656 (86.4)	50,259 (85.8)	
Race					
Black or African American	51,205 (22.4)	20,510 (19.5)	15,451 (24.0)	15,244 (26.0)	<0.001
White	153,910 (67.4)	74,174 (70.4)	42,810 (66.5)	36,926 (63.0)	
Other^a^ or Unknown	23,252 (10.2)	10,721 (10.2)	6134 (9.5)	6397 (10.9)	
Ethnicity					
Hispanic or Latinx	22,285 (9.8)	8393 (8.0)	6273 (9.7)	15,244 (26.0)	<0.001
Not Hispanic or Latinx	197,629 (86.5)	92,498 (87.8)	55,938 (86.9)	49,193 (84.0)	
Unknown	8453 (3.7)	4514 (4.3)	2184 (3.4)	1755 (3.0)	
Obese (BMI ≥ 35)	52,046 (22.8)	22,199 (21.1)	15,092 (23.4)	14,755 (25.2)	<0.001
Medical comorbidities					
Diabetes	79,090 (34.6)	39,358 (37.3)	21,702 (33.7)	18,030 (30.8)	<0.001
Cardiovascular disease	74,222 (32.5)	36,251 (34.4)	21,691 (33.7)	16,280 (27.8)	<0.001
Obstructive sleep apnea	73,127 (32.0)	26,271 (24.9)	22,163 (34.4)	24,693 (42.2)	<0.001
COPD	35,488 (15.5)	15,533 (14.7)	11,232 (17.4)	8723 (14.9)	<0.001
Cancer	40,769 (17.9)	20,260 (19.2)	11,500 (17.9)	9009 (15.4)	<0.001
Chronic kidney disease	31,693 (13.9)	16,276 (15.4)	9048 (14.1)	6369 (10.9)	<0.001
Liver disease	15,162 (6.6)	5446 (5.2)	5160 (8.0)	4556 (7.8)	<0.001
HIV	1845 (0.8)	621 (0.6)	756 (1.2)	468 (0.8)	<0.001
Smoking					
Current or former smoker	139,635 (61.1)	64,705 (61.4)	39,749 (61.7)	35,181 (60.1)	<0.001
Never smoker	88,732 (38.9)	40,700 (38.6)	24,646 (38.3)	23,386 (39.9)	

*COPD* chronic obstructive pulmonary disease, *HIV* human immunodeficiency virus, *VA* U.S. Department of Veteran Affairs.

^a^Other race includes American Indian Or Alaska Native, Asian, and Native Hawaiian Or Other Pacific Islander.

Compared to patients with no psychiatric disorders, patients with PTSD had a higher risk for both hospitalization and death following COVID-19 infection, adjusting for socio-demographics (Model 1; Table 2). Associations were attenuated but remained significant when additionally adjusting for medical comorbidities and smoking (Model 2). Patients with psychiatric disorders other than PTSD also showed a higher risk for hospitalization and death following COVID-19 infection in all models, relative to no psychiatric disorders. Associations with both hospitalization and death following COVID-19 infection were significantly higher for other psychiatric disorders than for PTSD in the full sample.

**Table 2:** Generalized linear models for associations of PTSD and other psychiatric disorders with 60-day hospitalization and death following COVID-19 infection (*n* = 228,367).

	Model 1		Model 2	
	RR (95% CI)	*p*-value	RR (95% CI)	*p*-value
Outcome: 60-day hospitalizations following COVID-19 infection (*n* = 34, 354 cases)				
PTSD	1.18 (1.15, 1.21)	<0.0001	1.09 (1.06, 1.12)	<0.0001
Other psychiatric disorders	1.34 (1.31, 1.37)	<0.0001	1.22 (1.19, 1.24)	<0.0001
No psychiatric disorders	Ref.	—	Ref.	—
PTSD vs. other psychiatric disorders	0.88 (0.86, 0.91)	<0.0001	0.90 (0.87, 0.92)	<0.0001
Outcome: 60-day deaths following COVID-19 infection (*n* = 13, 661 cases)				
PTSD	1.13 (1.08, 1.19)	<0.0001	1.08 (1.03, 1.13)	0.001
Other psychiatric disorders	1.23 (1.18, 1.27)	<0.0001	1.14 (1.10, 1.19)	<0.0001
No psychiatric disorders	Ref.	—	Ref.	—
PTSD vs. other psychiatric disorders	0.92 (0.88, 0.97)	0.002	0.94 (0.90, 0.99)	0.015

Model 1: age, age squared, sex, race, and ethnicity.

Model 2: Model 1 plus obese status, diabetes, cardiovascular disease including hypertension, obstructive sleep apnea, chronic obstructive pulmonary disease, cancer, chronic kidney disease, liver disease, human immunodeficiency virus, and smoking.

*RR* relative risk, *CI* confidence intervals, *PTSD* post-traumatic stress disorder.

As predicted, hospitalization and death following infection were strongly patterned by age, with both outcomes more likely among older individuals (e.g., 9.3% of those younger than 65 were hospitalized, while 21.6% of those 65 and older were hospitalized; 1.2% of those younger than 65 died, while 11.5% of those 65 and older died). Secondary analyses indicated that associations of psychiatric disorders with hospitalization and death following COVID-19 infection were evident across younger and older patients in models adjusted for socio-demographics (Table 3). Among older patients, psychiatric disorders other than PTSD had stronger associations with outcomes than PTSD. However, among younger patients, associations of PTSD and other psychiatric disorders with outcomes were of similar magnitude.

**Table 3:** Age-stratified generalized linear models for associations of PTSD and other psychiatric disorders with 60-day hospitalization and death following COVID-19 infection.

	Model 1		Model 2	
	RR (95% CI)	*p*-value	RR (95% CI)	*p*-value
Outcome: 60-day hospitalizations following COVID-19 infection				
Age < 65 (*n* = 122,049; *n* = 11,405 cases)				
PTSD	1.24 (1.18, 1.29)	<0.0001	1.16 (1.11, 1.21)	<0.0001
Other psychiatric disorders	1.28 (1.23, 1.34)	<0.0001	1.16 (1.12, 1.21)	<0.0001
No psychiatric disorders	Ref.	—	Ref.	—
PTSD vs. other psychiatric disorders	0.96 (0.92, 1.01)	0.104	1.00 (0.96, 1.04)	0.940
Age ≥ 65 (*n* = 106,318; *n* = 22,949 cases)				
PTSD	1.14 (1.11, 1.18)	<0.0001	1.06 (1.02, 1.09)	0.001
Other psychiatric disorders	1.37 (1.33, 1.40)	<0.0001	1.24 (1.21, 1.28)	<0.0001
No psychiatric disorders	Ref.	—	Ref.	—
PTSD vs. other psychiatric disorders	0.84 (0.81, 0.86)	<0.0001	0.85 (0.82, 0.88)	<0.0001
Outcome: 60-day deaths following COVID-19 infection				
Age < 65 (*n* = 122,049; *n* = 1429 cases)				
PTSD	1.14 (1.00, 1.31)	0.052	1.07 (0.94, 1.23)	0.305
Other psychiatric disorders	1.21 (1.08, 1.36)	0.001	1.08 (0.96, 1.22)	0.209
No psychiatric disorders	Ref.	—	Ref.	—
PTSD vs. other psychiatric disorders	0.94 (0.82, 1.08)	0.389	1.00 (0.87, 1.14)	0.944
Age ≥ 65 (*n* = 106,318; *n* = 12,232 cases)				
PTSD	1.12 (1.07, 1.17)	<0.0001	1.07 (1.02, 1.12)	0.004
Other psychiatric disorders	1.22 (1.17, 1.27)	<0.0001	1.15 (1.10, 1.19)	<0.0001
No psychiatric disorders	Ref.	—	Ref.	—
PTSD vs. other psychiatric disorders	0.92 (0.87, 0.97)	0.001	0.93 (0.89, 0.99)	0.012

Model 1: age, sex, race, and ethnicity.

Model 2: Model 1 plus obese status, diabetes, cardiovascular disease including hypertension, obstructive sleep apnea, chronic obstructive pulmonary disease, cancer, chronic kidney disease, liver disease, human immunodeficiency virus, and smoking.

*RR* relative risk, *CI* confidence intervals, *PTSD* post-traumatic stress disorder.

**Fig. 1:**
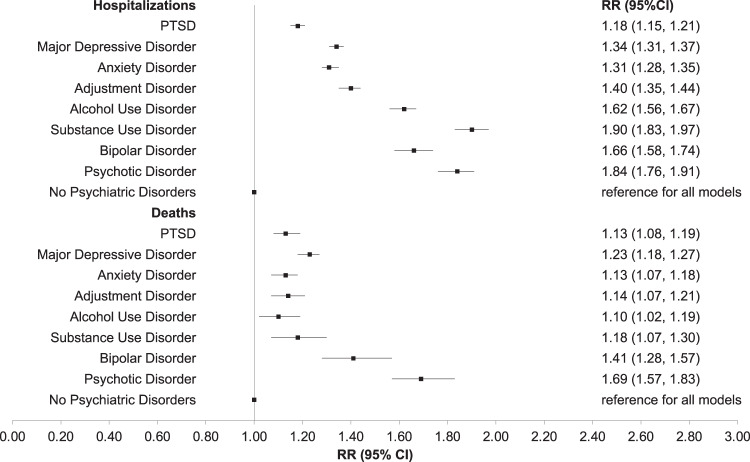
Relative risks of 60-day hospitalizations and deaths following COVID-19 infection for individual psychiatric disorders. RR relative risk, CI confidence intervals, PTSD post-traumatic stress disorder. Reference group for each model is No Psychiatric Disorders (*n* = 105,405 (46.2%)); each individual psychiatric disorder was estimated in a separate model as the primary predictor and adjusted for age, age squared, sex, race, and ethnicity.

When examining associations for any psychiatric disorder (including PTSD) compared to no psychiatric disorders, we found 27% increased risk for hospitalization (socio-demographic adjusted RR = 1.27, 95% CI 1.24–1.29; fully adjusted RR = 1.16, 95% CI 1.14–1.19) and 19% increased risk for death (socio-demographic adjusted RR = 1.19, 95% CI 1.15–1.23; fully adjusted RR = 1.12, 95% CI 1.08–1.16) associated with any versus no psychiatric disorder. Delving into individual psychiatric disorders, all individual psychiatric disorders that we assessed were each associated with a higher risk for hospitalization and death as compared to no disorders, adjusted for socio-demographics (Table 4**;** Fig. 1). Effect sizes were generally similar across all psychiatric disorders indicating that the presence of any psychiatric disorder is a risk factor for adverse outcomes of COVID-19. Age-stratified models for individual psychiatric disorders indicated particularly strong effect estimates for substance use and psychotic disorders on risk for hospitalization, and of psychotic disorders on risk for death, in both younger and older veterans (Table S2). Associations between PTSD and increased risk for hospitalizations appeared to be driven by strong associations among the youngest veterans (<40 years old) (Table S3). When considering diagnoses of PTSD or adjustment disorder together, the pattern of associations did not change (Table S4). Lastly, findings were nearly identical when excluding those with breakthrough infections (Table S5).

**Table 4:** Individual generalized linear models for associations between each psychiatric disorder with 60-day hospitalization and death following COVID-19 infection (*n* = 228,367).

		Model 1		Model 2	
	No. (%)	RR (95% CI)	*p*-value	RR (95% CI)	*p*-value
Outcome: 60-day hospitalizations following COVID-19 infection					
PTSD	58,567 (25.6)	1.18 (1.15, 1.21)	<0.0001	1.09 (1.06, 1.12)	<0.0001
Other psychiatric disorders	64,395 (28.2)	1.34 (1.31, 1.37)	<0.0001	1.22 (1.19, 1.24)	<0.0001
Major depressive disorder	78,724 (34.5)	1.34 (1.31, 1.37)	<0.0001	1.21 (1.18, 1.23)	<0.0001
Other psychiatric disorders	44,238 (19.4)	1.15 (1.12, 1.18)	<0.0001	1.09 (1.06, 1.12)	<0.0001
Anxiety disorder	51,815 (22.7)	1.31 (1.28, 1.35)	<0.0001	1.19 (1.16, 1.22)	<0.0001
Other psychiatric disorders	71,147 (31.2)	1.25 (1.22, 1.27)	<0.0001	1.15 (1.12, 1.17)	<0.0001
Adjustment disorder	29,259 (12.8)	1.40 (1.35, 1.44)	<0.0001	1.26 (1.22, 1.30)	<0.0001
Other psychiatric disorders	93,703 (41.0)	1.24 (1.21, 1.26)	<0.0001	1.14 (1.12, 1.16)	<0.0001
Alcohol use disorder	21,885 (9.6)	1.62 (1.56, 1.67)	<0.0001	1.45 (1.40, 1.49)	<0.0001
Other psychiatric disorders	101,077 (44.3)	1.21 (1.18, 1.23)	<0.0001	1.11 (1.09, 1.13)	<0.0001
Substance use disorder	13,440 (5.9)	1.90 (1.83, 1.97)	<0.0001	1.62 (1.56, 1.68)	<0.0001
Other psychiatric disorders	109,522 (48.0)	1.20 (1.18, 1.23)	<0.0001	1.11 (1.09, 1.14)	<0.0001
Bipolar disorder	8791 (3.8)	1.66 (1.58, 1.74)	<0.0001	1.46 (1.39, 1.53)	<0.0001
Other psychiatric disorders	114,171 (50.0)	1.25 (1.22, 1.27)	<0.0001	1.14 (1.12, 1.17)	<0.0001
Psychotic disorder	7254 (3.2)	1.84 (1.76, 1.91)	<0.0001	1.66 (1.59, 1.73)	<0.0001
Other psychiatric disorders	115,708 (50.7)	1.22 (1.20, 1.25)	<0.0001	1.12 (1.10, 1.15)	<0.0001
Outcome: 60-day deaths following COVID-19 infection					
PTSD	58,567 (25.6)	1.13 (1.08, 1.19)	<0.0001	1.08 (1.03, 1.13)	0.001
Other psychiatric disorders	64,395 (28.2)	1.23 (1.18, 1.27)	<0.0001	1.14 (1.10, 1.19)	<0.0001
Major depressive disorder	78,724 (34.5)	1.23 (1.18, 1.27)	<0.0001	1.13 (1.09, 1.17)	<0.0001
Other psychiatric disorders	44,238 (19.4)	1.14 (1.09, 1.19)	<0.0001	1.10 (1.05, 1.15)	<0.0001
Anxiety disorder	51,815 (22.7)	1.13 (1.07, 1.18)	<0.0001	1.06 (1.01, 1.11)	0.028
Other psychiatric disorders	71,147 (31.2)	1.22 (1.18, 1.26)	<0.0001	1.15 (1.11, 1.19)	<0.0001
Adjustment disorder	29,259 (12.8)	1.14 (1.07, 1.21)	<0.0001	1.05 (0.99, 1.12)	0.102
Other psychiatric disorders	93,703 (41.0)	1.20 (1.16, 1.24)	<0.0001	1.13 (1.10, 1.17)	<0.0001
Alcohol use disorder	21,885 (9.6)	1.10 (1.02, 1.19)	0.001	1.03 (0.95, 1.11)	0.482
Other psychiatric disorders	101,077 (44.3)	1.20 (1.16, 1.24)	<0.0001	1.13 (1.09, 1.17)	<0.0001
Substance use disorder	13,440 (5.9)	1.18 (1.07, 1.30)	<0.0001	1.04 (0.94, 1.14)	0.476
Other psychiatric disorders	109,522 (48.0)	1.19 (1.15, 1.23)	<0.0001	1.12 (1.09, 1.16)	<0.0001
Bipolar disorder	8791 (3.8)	1.41 (1.28, 1.57)	<0.0001	1.29 (1.17, 1.43)	<0.0001
Other psychiatric disorders	114,171 (50.0)	1.18 (1.14, 1.22)	<0.0001	1.11 (1.07, 1.15)	<0.0001
Psychotic disorder	7254 (3.2)	1.69 (1.57, 1.83)	<0.0001	1.58 (1.46, 1.70)	<0.0001
Other psychiatric disorders	115,708 (50.7)	1.15 (1.11, 1.19)	<0.0001	1.08 (1.05, 1.12)	<0.0001

Reference group for each model is No Psychiatric Disorders (*n* = 105,405 (46.2%)).

Model 1: age, age squared, sex, race, and ethnicity.

Model 2: Model 1 plus obese status, diabetes, cardiovascular disease including hypertension, obstructive sleep apnea, chronic obstructive pulmonary disease, cancer, chronic kidney disease, liver disease, human immunodeficiency virus, and smoking.

*RR* relative risk, *CI* confidence intervals, *PTSD* post-traumatic stress disorder.

## Discussion

Among 228,367 VA patients, PTSD and other psychiatric disorders were associated with a significantly higher risk for hospitalization and death in the 60 days following a positive test for COVID-19, compared to no psychiatric disorders. Adjusting for medical comorbidities attenuated, but did not fully account for, associations of PTSD and other psychiatric disorders with increased risk for hospitalization and death following COVID-19 infection, indicating that comorbidities may contribute to some but not all of the increased risks. Although PTSD increased the risk for COVID-19 hospitalization and death across the full sample, there were some differences by age. Among patients under age 65, PTSD and other psychiatric conditions increased the risk for adverse COVID-19 outcomes relative to those without psychiatric conditions, with similar effect sizes for PTSD and other psychiatric disorders. However, among patients aged 65 and older, other psychiatric conditions were more strongly associated with adverse outcomes than PTSD. All the other psychiatric disorders we examined in our study were associated with increased risk for COVID-19 hospitalization and death, with substance and alcohol use, bipolar, and psychotic disorders showing particularly strong associations with hospitalizations, and psychotic disorders showing particularly strong associations with deaths. Overall, our data add to an accumulating evidence base that underscores the need to consider patients with psychiatric disorders, including PTSD, as a vulnerable group in the context of the COVID-19 pandemic.

The present study adds PTSD to the list of psychiatric disorders that increase the risk for adverse outcomes of COVID-19 [4–6, 28]. In our sample of VA patients, PTSD and other psychiatric disorders increased the risk for poorer COVID-19 outcomes across younger and older patient groups, suggesting that psychiatric disorders increase the risk for adverse COVID-19 sequelae across adulthood. This elevated risk remained even when accounting for medical comorbidities and smoking, indicating that PTSD could influence COVID-19-related outcomes via additional or unadjusted mechanisms such as behaviors we did not assess (e.g., alcohol consumption, sleep, and COVID-19-related behaviors), dysregulation of biological systems (e.g., elevated inflammation and dysregulated immune functioning), or increased somatization or anxiety (e.g., heightened somatic symptoms or anxiety related to COVID-19 symptoms). Psychological stress from the pandemic overall or the experience of SARS-Cov-2 infection itself may have exacerbated psychiatric symptoms, which could increase circulating inflammatory cytokines, catecholamines, and glucocorticoids consistent with the concept of elevated allostatic load [29], which may, in turn, have contributed to increased risk of hospitalizations or deaths. Adjustment disorder, emotional or behavioral symptoms in response to a stressor within three months of stressor onset, may have mechanisms of increased risk that overlap with those of PTSD, though sensitivity analyses suggest moderate comorbidity and that estimates considering either diagnosis indicate elevated risk for poor COVID-19 outcomes. As we could not discern the traumas or stressors contributing to these diagnoses, it is difficult to determine their distinct and overlapping effects.

Contrary to our initial hypothesis, PTSD did not confer a higher risk for adverse outcomes than other psychiatric disorders. The highest risks were associated with substance and alcohol use, bipolar, and psychotic disorders, conditions that have also increased the risk for adverse COVID-19 sequelae in prior studies [4, 30]. While reasons for the differences in effect sizes across disorders remain unclear, physiological effects of drugs of abuse, increased likelihood of homelessness, or other psychosocial adversities [30, 31] could be explaining excess risk. Indeed, among patients aged 65 and older, those with PTSD had a lower risk for adverse outcomes of COVID-19 compared to those with other psychiatric disorders. Specific biological or psychosocial mechanisms by which other psychiatric disorders increased risk may have interacted with age, such that these factors particularly increased risk in older veterans. Furthermore, chronicity of PTSD may influence outcomes. If younger compared to older veterans are earlier in the course of their PTSD illness, they may have different patterns of biological dysregulation, which in turn could influence the risk for adverse outcomes of COVID-19. Future studies should gather data on the chronicity of PTSD and examine if this influences outcomes. Among younger patients, PTSD and other psychiatric disorders conferred increases in the risk of similar magnitude. These findings for outcomes of an infectious disease are in contrast to prior work focusing on chronic physical diseases in US VA patients, which has suggested that individuals with PTSD have a higher risk for chronic diseases than patients with psychiatric disorders other than PTSD (e.g., autoimmune disease, hypertension, dyslipidemia) [7, 32, 33].

Overall, veterans with psychiatric disorder diagnoses had 27% increased risk for hospitalization and 19% increased risk for death following a positive COVID-19 test. Each psychiatric disorder that we assessed individually was associated with a significantly elevated risk for hospitalizations and deaths following COVID-19. Prior studies similarly indicated an elevated risk for poor COVID-19 sequelae in individuals with psychiatric disorders [4–6], suggesting that poor mental health may be an important risk factor for adverse COVID-19 outcomes. In our sample, associations largely held when accounting for medical comorbidities and smoking, in contrast with a recent study of 15,168 French adults hospitalized for COVID-19 in which associations between psychiatric conditions and COVID-19 mortality were fully explained by adjustment for obesity, count of medical conditions, and medication use [34]. Differences in the sample population (e.g., French adults hospitalized for COVID-19, 5.7% of whom had a psychiatric condition, versus VA patients with positive SARS-CoV-2 tests, 53.8% of whom had a psychiatric diagnosis) and the adjustment for medication use might explain these disparate findings. Future studies should examine mechanisms underlying the differing magnitude of association of specific psychiatric diagnoses with poor COVID-19 outcomes, and examine medication type as a potential moderator or mediator of effects. However, findings in the literature to date indicate that multiple different psychiatric disorders should be considered as potential vulnerability factors for adverse outcomes of COVID-19 across all disorders and age groups.

Our study benefited from a very large sample of patients with comprehensive healthcare data and a high prevalence of psychiatric diagnoses. However, there are several limitations. First, selection biases may have occurred due to including only patients who had records of positive SARS-CoV-2 tests within the VA system, excluding patients who had a positive SARS-CoV-2 test that was not recorded in VA, were asymptomatic and untested, completed a home test (relatively less common during 2020 and 2021 compared to 2022), and did not have access to tests. However, requiring that patients accessed VA care in the year prior to their SARS-CoV-2 test may lower concerns of differential healthcare access. Second, it is possible that some hospitalizations in the 60 days following a positive SARS-CoV-2 test were not specifically due to COVID-19 illness, though we excluded admissions without isolation measures or for multiple alternative reasons (e.g., admissions to nursing homes or other long-term care facilities, psychiatric or spinal cord injury admissions). Third, while we adjusted for basic socio-demographics and medical comorbidities known to impact COVID-19 outcomes, the clinical record data source limited our ability to richly characterize covariates, including important social determinants of health. Moreover, there is evidence of both over and underdiagnosis of psychiatric conditions in clinical records [35], potentially leading to misclassification of our exposures. Finally, our sample included only VA patients who accessed VA healthcare during the COVID-19 pandemic, and almost 90% of the sample was men who may be at higher risk for more severe COVID-19 compared to women [36]; therefore, additional studies are needed to examine if findings generalize to different populations. Follow-up studies should also determine the specific mechanisms that underlie increased risk for COVID-19 hospitalizations or death associated with PTSD and other psychiatric disorders.

The U.S. Centers for Disease Control and Prevention (CDC) added mood and schizophrenia spectrum disorders to their list of medical conditions that may increase the likelihood of severe COVID-19 illness in October 2021 [37]. Our data indicate that PTSD and other psychiatric disorders should be considered for this list as well. As stressors and trauma related to the ongoing global COVID-19 pandemic may exacerbate or elicit PTSD symptoms [38], the effects of PTSD on COVID-19 prognosis may become even more salient. As PTSD and other psychiatric diagnoses may result in worse COVID-19 outcomes, mental health practitioners can play a key role in COVID-19 prevention and vaccine advocacy for their patients [39]. Similarly, clinicians in general medicine could screen for psychiatric comorbidities to identify individuals at higher risk for hospitalization and death following COVID-19. Accumulating data linking psychiatric disorders with COVID-19 disease outcomes underscore the need to coordinate across disciplinary boundaries to improve public health in the context of the COVID-19 pandemic.

## Supplementary information

Supplement

## References

[1] WHO. WHO Coronavirus (COVID-19) Dashboard. 2022. https://covid19.who.int. Accessed 13 June 2022.

[2] Bauer ME, Teixeira AL (2019). Inflammation in psychiatric disorders: what comes first?. Ann N Y Acad Sci.

[3] Goodell S, Druss BG, Walker ER. 2011. Mental Disorders and Medical Comorbidity. Robert Wood JohnsonFoundation: Princeton, NJ, USA. Available at: http://www.integration.samhsa.gov/workforce/mental_disorders_and_medical_comorbidity.pdf.

[4] Fond G, Nemani K, Etchecopar-Etchart D, Loundou A, Goff DC, Lee SW (2021). Association between mental health disorders and mortality among patients with COVID-19 in 7 countries: a systematic review and meta-analysis. JAMA Psychiatry.

[5] Vai B, Mazza MG, Colli CD, Foiselle M, Allen B, Benedetti F (2021). Mental disorders and risk of COVID-19-related mortality, hospitalisation, and intensive care unit admission: a systematic review and meta-analysis. Lancet Psychiatry.

[6] Toubasi AA, AbuAnzeh RB, Tawileh HBA, Aldebei RH, Alryalat SAS (2021). A meta-analysis: the mortality and severity of COVID-19 among patients with mental disorders. Psychiatry Res.

[7] O’Donovan A, Cohen BE, Seal KH, Bertenthal D, Margaretten M, Nishimi K (2015). Elevated risk for autoimmune disorders in Iraq and Afghanistan veterans with posttraumatic stress disorder. Biol Psychiatry.

[8] Edmondson D, Kronish IM, Shaffer JA, Falzon L, Burg MM (2013). Posttraumatic stress disorder and risk for coronary heart disease: a meta-analytic review. Am Heart J.

[9] Sumner JA, Nishimi KM, Koenen KC, Roberts AL, Kubzansky LD (2020). Posttraumatic stress disorder and inflammation: Untangling issues of bidirectionality. Biol Psychiatry.

[10] van den Berk-Clark C, Secrest S, Walls J, Hallberg E, Lustman PJ, Schneider FD (2018). Association between posttraumatic stress disorder and lack of exercise, poor diet, obesity, and co-occuring smoking: a systematic review and meta-analysis. Health Psychol.

[11] O’Donovan A, Sun B, Cole S, Rempel H, Lenoci M, Pulliam L (2011). Transcriptional control of monocyte gene expression in post-traumatic stress disorder. Dis Markers.

[12] O’Donovan A, Slavich GM, Epel ES, Neylan TC (2013). Exaggerated neurobiological sensitivity to threat as a mechanism linking anxiety with increased risk for diseases of aging. Neurosci Biobehav Rev.

[13] Li X, Wang J, Zhou J, Huang P, Li J (2017). The association between post-traumatic stress disorder and shorter telomere length: a systematic review and meta-analysis. J Affect Disord.

[14] Lamontagne SJ, Pizzagalli DA, Olmstead MC (2021). Does inflammation link stress to poor COVID-19 outcome?. Stress Health.

[15] Hu B, Huang S, Yin L (2021). The cytokine storm and COVID-19. J Med Virol.

[16] Barsky AJ, Orav EJ, Bates DW (2005). Somatization increases medical utilization and costs independent of psychiatric and medical comorbidity. Arch Gen Psychiatry.

[17] O’Donnell CJ, Longacre LS, Cohen BE, Fayad ZA, Gillespie CF, Liberzon I (2021). Posttraumatic stress disorder and cardiovascular disease: state of the science, knowledge gaps, and research opportunities. JAMA Cardiol.

[18] Maguen S, Madden E, Cohen B, Bertenthal D, Neylan T, Talbot L (2013). The relationship between body mass index and mental health among iraq and afghanistan veterans. J Gen Intern Med.

[19] Renna ME, O’Toole MS, Spaeth PE, Lekander M, Mennin DS (2018). The association between anxiety, traumatic stress, and obsessive-compulsive disorders and chronic inflammation: a systematic review and meta-analysis. Depress Anxiety.

[20] Yehuda R, Hoge CW, McFarlane AC, Vermetten E, Lanius RA, Nievergelt CM (2015). Post-traumatic stress disorder. Nat Rev Dis Prim.

[21] Haderlein TP, Wong MS, Yuan A, Llorente MD, Washington DL (2021). Association of PTSD with COVID-19 testing and infection in the Veterans Health Administration. J Psychiatr Res.

[22] Cardemil CV, Dahl R, Prill MM, Cates J, Brown S, Perea A (2021). COVID-19-related hospitalization rates and severe outcomes among veterans from 5 Veterans Affairs Medical Centers: hospital-based surveillance study. JMIR Public Health Surveill.

[23] Ioannou GN, Locke E, Green P, Berry K, O’Hare AM, Shah JA, et al. Risk factors for hospitalization, mechanical ventilation, or death among 10 131 US veterans with SARS-CoV-2 infection. JAMA Netw Open. 2020;3:e2022310. https://doi.org/jamanetworkopen.2020.22310. 10.1001/jamanetworkopen.2020.22310 PMC7512055 32965502

[24] Cohn BA, Cirillo PM, Murphy CC, Krigbaum NY, Wallace AW (2021). SARS-CoV-2 vaccine protection and deaths among US veterans during 2021. Science.

[25] Kelly JD, Bravata DM, Bent S, Wray CM, Leonard SJ, Boscardin WJ (2021). Association of social and behavioral risk factors with mortality among US veterans with COVID-19. JAMA Netw Open.

[26] Zou G (2004). A modified poisson regression approach to prospective studies with binary data. Am J Epidemiol.

[27] CDC. Cases, Data, and Surveillance. Centers for Disease Control and Prevention. 2020. https://www.cdc.gov/coronavirus/2019-ncov/covid-data/investigations-discovery/hospitalization-death-by-age.html. Accessed 30 Aug 2021.

[28] Ceban F, Nogo D, Carvalho IP, Lee Y, Nasri F, Xiong J (2021). Association between mood disorders and risk of COVID-19 infection, hospitalization, and death: a systematic review and meta-analysis. JAMA Psychiatry.

[29] McEwen BS (2005). Stressed or stressed out: what is the difference?. J Psychiatry Neurosci.

[30] Wang QQ, Kaelber DC, Xu R, Volkow ND (2021). COVID-19 risk and outcomes in patients with substance use disorders: analyses from electronic health records in the United States. Mol Psychiatry.

[31] Leifheit KM, Chaisson LH, Medina JA, Wahbi RN, Shover CL (2021). Elevated mortality among people experiencing homelessness with COVID-19. Open Forum Infect Dis.

[32] Cohen BE, Marmar C, Ren L, Bertenthal D, Seal KH (2009). Association of cardiovascular risk factors with mental health diagnoses in Iraq and Afghanistan war veterans using VA health care. JAMA.

[33] Cohen BE, Gima K, Bertenthal D, Kim S, Marmar CR, Seal KH (2010). Mental health diagnoses and utilization of VA non-mental health medical services among returning Iraq and Afghanistan veterans. J Gen Intern Med.

[34] Hoertel N, Sánchez-Rico M, de la Muela P, Abellán M, Blanco C, Leboyer M, et al. Risk of death in individuals hospitalized for COVID-19 with and without psychiatric disorders: an observational multicenter study in France. Biol Psychiatry Glob Open Sci. 2022. 10.1016/j.bpsgos.2021.12.007. 10.1016/j.bpsgos.2021.12.007 PMC8730644 35013734

[35] Holowka DW, Marx BP, Gates MA, Litman HJ, Ranganathan G, Rosen RC (2014). PTSD diagnostic validity in Veterans Affairs electronic records of Iraq and Afghanistan veterans. J Consult Clin Psychol.

[36] Gomez JMD, Du-Fay-de-Lavallaz J, Fugar S, Sarau A, Simmons JA, Clark B (2021). Sex differences in COVID-19 hospitalization and mortality. J Women’s Health.

[37] CDC. COVID-19 and Your Health. Centers for Disease Control and Prevention. 2020. https://www.cdc.gov/coronavirus/2019-ncov/need-extra-precautions/people-with-medical-conditions.html. Accessed 17 Oct 2021.

[38] Yuan K, Gong YM, Liu L, Sun YK, Tian SS, Wang YJ (2021). Prevalence of posttraumatic stress disorder after infectious disease pandemics in the twenty-first century, including COVID-19: a meta-analysis and systematic review. Mol Psychiatry.

[39] Goldberg JF (2021). How should psychiatry respond to COVID-19 anti-vax attitudes?. J Clin Psychiatry.

