# Supplementary material for: Post-traumatic stress disorder and risk for hospitalization and death following COVID-19 infection
Source: Transl Psychiatry. 2022 Nov 22;12:482. doi: 10.1038/s41398-022-02156-w (PMC9678873; doi:10.1038/s41398-022-02156-w)
Supplement: Supplementary file 1 — Supplement [file 41398_2022_2156_MOESM1_ESM.docx]

**SUPPLEMENT**

**Figure S1. Flowchart of exclusions for deriving the final analytic sample.**

**
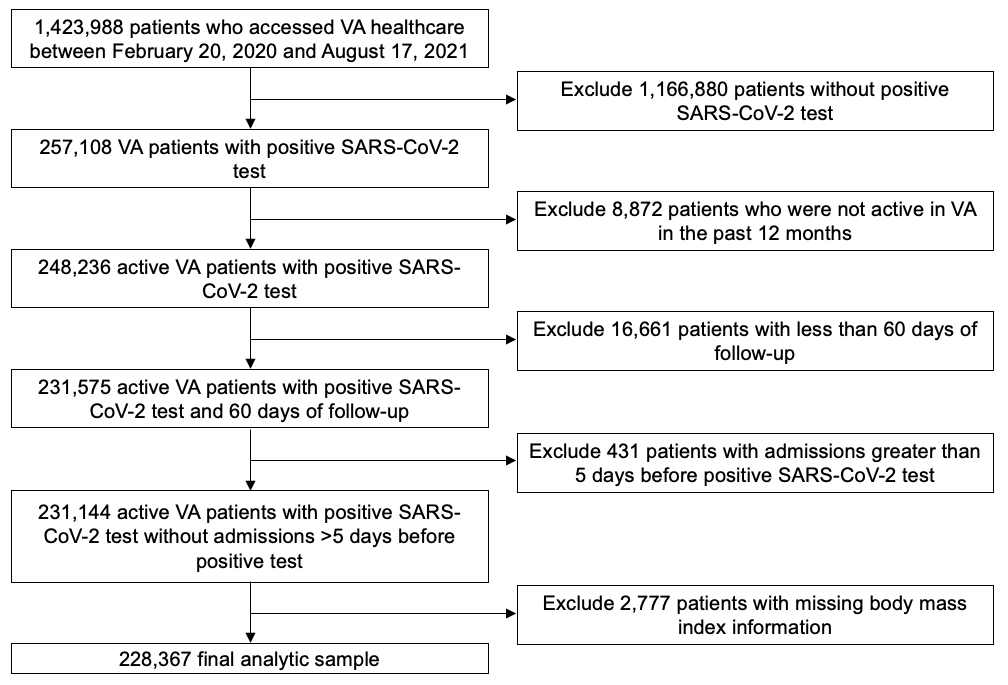
**

| **Table S1. International Classification of Diseases, Ninth Revision Clinical Modification (ICD-9-CM) and ICD-10-CM codes used to define psychiatric disorders** | | |
| --- | --- | --- |
| **Psychiatric Disorder** | **ICD-9-CM** | **ICD-10-CM Codes** |
| Posttraumatic Stress Disorder | 309.81 | F43.1 |
| Major Depressive Disorder | 293.83, 296.20-296.25, 296.30-296.35, 296.82, 300.4, 311 | F32 (excluding F32.5), F33 (excluding F33.40, F33.42), F34.1, F34.8, F34.9, F39 |
| Anxiety Disorder | 300.00-300.09, 300.20-300.29, 300.3, 300.7, 300.81-300.89 | F40, F41, F42, F45 (excluding F45.41, F45.42), F63.3 |
| Substance Use Disorder | 304 (excluding 304.03, 304,13, 304.23, 304.33, 304.43, 304.53, 304.63, 304.73, 304.83, 304.93), 305.20-305.92 (excluding 305.23, 305.33, 305.43, 305.53, 305.63, 305.73, 305.83) | F11.1, F11.2 (excluding F11.21), F12.1, F12.2 (excluding F12.21), F13.1, F13.2 (excluding F13.21), F14.1, F14.2 (excluding F14.21), F15.1, F15.2 (excluding F15.21), F16.1, F16.2 (excluding F16.21), F18.1, F18.2 (excluding F18.21), F19.1, F19.2 (excluding F19.21) |
| Alcohol Use Disorder | 303 (excluding 303.03, 303.93), 305.00-305.02 | F10.1, F10.2 (excluding F10.21) |
| Bipolar Disorder | 296.00-296.15 (excluding 296.06), 296.40-296.81 (excluding 296.46, 296.56, 296.66), 296.89, 301.13 | F30 (excluding F304), F31 (excluding F31.70, F31.72, F31.74, F31.76, F31.78), F34.0 |
| Psychotic Disorder | 295 (excluding 295.05, 295.15, 295.25, 295.35, 295.45, 295.55, 295.65, 295.75, 295,85, 295.95), 297, 298 | F20, F21, F22, F23, F24, F25, F28, F29 |
| Eating Disorder | 307.1, 307.50, 307.51 | F50 |
| Attention-Deficit Hyperactivity Disorder | 314.00, 314.01 | F90 |
| Dissociative Disorder | 300.11-300.19, 300.6 | F44, F48.1 |
| Adjustment Disorder | 308, 309 (excluding 309.81) | F43.2, F43.8, F43.9 |

| **Table S2.** **Age-stratified generalized linear models for associations between each psychiatric disorder with 60-day hospitalization and death following COVID-19 infection** | | | | | |
| --- | --- | --- | --- | --- | --- |
|  |  | **Model 1** | | **Model 2** | |
|  | *N (%)* | *RR (95% CI)* | *p-value* | *RR (95% CI)* | *p-value* |
| **Outcome: 60-day Hospitalizations following COVID-19 Infection** | | | | | |
| *Age <65 (n=122,049)* |  |  |  |  |  |
| PTSD | 38,638 (36.3) | 1.24 (1.18, 1.29) | <.0001 | 1.16 (1.11, 1.21) | <.0001 |
| Other Psychiatric Disorders | 39,094 (32.0) | 1.28 (1.23, 1.34) | <.0001 | 1.16 (1.12, 1.21) | <.0001 |
|  |  |  |  |  |  |
| Major Depressive Disorder | 51,796 (48.7) | 1.35 (1.30, 1.41) | <.0001 | 1.22 (1.18, 1.27) | <.0001 |
| Other Psychiatric Disorders | 25,936 (21.3) | 1.08 (1.03, 1.14) | 0.002 | 1.04 (0.99, 1.27) | 0.131 |
|  |  |  |  |  |  |
| Anxiety Disorder | 37,504 (35.3) | 1.33 (1.27, 1.38) | <.0001 | 1.21 (1.16, 1.26) | <.0001 |
| Other Psychiatric Disorders | 40,228 (33.0) | 1.21 (1.17, 1.27) | <.0001 | 1.13 (1.08, 1.17) | <.0001 |
|  |  |  |  |  |  |
| Adjustment Disorder | 21,524 (20.2) | 1.38 (1.32, 1.45) | <.0001 | 1.25 (1.19, 1.32) | <.0001 |
| Other Psychiatric Disorders | 56,208 (46.1) | 1.22 (1.17, 1.27) | <.0001 | 1.13 (1.09, 1.17) | <.0001 |
|  |  |  |  |  |  |
| Alcohol Use Disorder | 16,037 (15.1) | 1.69 (1.61, 1.77) | <.0001 | 1.52 (1.45, 1.60) | <.0001 |
| Other Psychiatric Disorders | 61,695 (50.5) | 1.14 (1.10, 1.19) | <.0001 | 1.06 (1.02, 1.10) | 0.003 |
|  |  |  |  |  |  |
| Substance Use Disorder | 10,054 (9.5) | 1.99 (1.89, 2.10) | <.0001 | 1.72 (1.63, 1.81) | <.0001 |
| Other Psychiatric Disorders | 67,678 (55.5) | 1.13 (1.09, 1.18) | <.0001 | 1.06 (1.02, 1.10) | 0.003 |
|  |  |  |  |  |  |
| Bipolar Disorder | 6,435 (6.1) | 1.69 (1.58, 1.82) | <.0001 | 1.46 (1.36, 1.57) | <.0001 |
| Other Psychiatric Disorders | 71,297 (58.4) | 1.22 (1.18, 1.27) | <.0001 | 1.14 (1.09, 1.18) | <.0001 |
|  |  |  |  |  |  |
| Psychotic Disorder | 3,699 (3.5) | 2.02 (1.88, 2.18) | <.0001 | 1.71 (1.59, 1.84) | <.0001 |
| Other Psychiatric Disorders | 74,033 (60.7) | 1.21 (1.17, 1.26) | <.0001 | 1.12 (1.08, 1.16) | <.0001 |
|  |  |  |  |  |  |
| *Age ≥65 (n=106,318)* |  |  |  |  |  |
| PTSD | 19,916 (18.7) | 1.14 (1.11, 1.18) | <.0001 | 1.06 (1.02, 1.09) | 0.001 |
| Other Psychiatric Disorders | 25,271 (20.7) | 1.37 (1.33, 1.40) | <.0001 | 1.24 (1.21, 1.28) | <.0001 |
|  |  |  |  |  |  |
| Major Depressive Disorder | 26,909 (25.3) | 1.33 (1.29, 1.36) | <.0001 | 1.19 (1.16, 1.23) | <.0001 |
| Other Psychiatric Disorders | 18,278 (15.0) | 1.18 (1.15, 1.22) | <.0001 | 1.12 (1.09, 1.16) | <.0001 |
|  |  |  |  |  |  |
| Anxiety Disorder | 14,299 (13.4) | 1.29 (1.25, 1.34) | <.0001 | 1.17 (1.14, 1.21) | <.0001 |
| Other Psychiatric Disorders | 30,888 (25.3) | 1.26 (1.23, 1.29) | <.0001 | 1.16 (1.13, 1.19) | <.0001 |
|  |  |  |  |  |  |
| Adjustment Disorder | 7,723 (7.3) | 1.40 (1.35, 1.46) | <.0001 | 1.25 (1.20, 1.30) | <.0001 |
| Other Psychiatric Disorders | 37,464 (30.7) | 1.24 (1.21, 1.27) | <.0001 | 1.15 (1.12, 1.17) | <.0001 |
|  |  |  |  |  |  |
| Alcohol Use Disorder | 5,847 (5.5) | 1.50 (1.43, 1.56) | <.0001 | 1.34 (1.28, 1.40) | <.0001 |
| Other Psychiatric Disorders | 39,340 (32.2) | 1.24 (1.21, 1.27) | <.0001 | 1.14 (1.11, 1.17) | <.0001 |
|  |  |  |  |  |  |
| Substance Use Disorder | 3,385 (3.2) | 1.69 (1.61, 1.78) | <.0001 | 1.44 (1.37, 1.52) | <.0001 |
| Other Psychiatric Disorders | 41,802 (34.3) | 1.24 (1.21, 1.27) | <.0001 | 1.14 (1.12, 1.17) | <.0001 |
|  |  |  |  |  |  |
| Bipolar Disorder | 2,356 (2.2) | 1.59 (1.49, 1.70) | <.0001 | 1.42 (1.33, 1.52) | <.0001 |
| Other Psychiatric Disorders | 42,831 (35.1) | 1.25 (1.22, 1.28) | <.0001 | 1.15 (1.13, 1.18) | <.0001 |
|  |  |  |  |  |  |
| Psychotic Disorder | 3,552 (3.3) | 1.71 (1.63, 1.80) | <.0001 | 1.59 (1.52, 1.67) | <.0001 |
| Other Psychiatric Disorders | 41,635 (34.1) | 1.23 (1.20, 1.26) | <.0001 | 1.13 (1.10, 1.15) | <.0001 |
|  |  |  |  |  |  |
| **Outcome: 60-day Deaths following COVID-19 Infection** | | | | | |
| *Age <65 (n=122,049)* |  |  |  |  |  |
| PTSD | 38,638 (36.3) | 1.14 (1.00, 1.31) | 0.052 | 1.07 (0.94, 1.23) | 0.305 |
| Other Psychiatric Disorders | 39,094 (32.0) | 1.21 (1.08, 1.36) | 0.001 | 1.08 (0.96, 1.22) | 0.209 |
|  |  |  |  |  |  |
| Major Depressive Disorder | 51,796 (48.7) | 1.21 (1.08, 1.36) | 0.001 | 1.07 (0.95, 1.20) | 0.289 |
| Other Psychiatric Disorders | 25,936 (21.3) | 1.14 (0.99, 1.31) | 0.069 | 1.10 (0.95, 1.27) | 0.185 |
|  |  |  |  |  |  |
| Anxiety Disorder | 37,504 (35.3) | 1.12 (0.98, 1.29) | 0.090 | 1.02 (0.89, 1.17) | 0.821 |
| Other Psychiatric Disorders | 40,228 (33.0) | 1.23 (1.09, 1.38) | <.0001 | 1.12 (0.99, 1.26) | 0.067 |
|  |  |  |  |  |  |
| Adjustment Disorder | 21,524 (20.2) | 1.17 (1.00, 1.37) | 0.048 | 1.03 (0.88, 1.21) | 0.688 |
| Other Psychiatric Disorders | 56,208 (46.1) | 1.19 (1.06, 1.33) | 0.002 | 1.09 (0.98, 1.22) | 0.126 |
|  |  |  |  |  |  |
| Alcohol Use Disorder | 16,037 (15.1) | 1.19 (1.01, 1.40) | 0.035 | 1.09 (0.93, 1.29) | 0.282 |
| Other Psychiatric Disorders | 61,695 (50.5) | 1.18 (1.06, 1.32) | 0.003 | 1.07 (0.96, 1.20) | 0.223 |
|  |  |  |  |  |  |
| Substance Use Disorder | 10,054 (9.5) | 1.16 (0.97, 1.40) | 0.107 | 0.98 (0.81, 1.18) | 0.827 |
| Other Psychiatric Disorders | 67,678 (55.5) | 1.19 (1.07, 1.33) | 0.002 | 1.10 (0.98, 1.23) | 0.101 |
|  |  |  |  |  |  |
| Bipolar Disorder | 6,435 (6.1) | 1.39 (1.10, 1.75) | 0.006 | 1.18 (0.93, 1.49) | 0.168 |
| Other Psychiatric Disorders | 71,297 (58.4) | 1.17 (1.05, 1.30) | 0.005 | 1.07 (0.96, 1.19) | 0.235 |
|  |  |  |  |  |  |
| Psychotic Disorder | 3,699 (3.5) | 1.70 (1.36, 2.12) | <.0001 | 1.40 (1.12, 1.75) | 0.003 |
| Other Psychiatric Disorders | 74,033 (60.7) | 1.14 (1.03, 1.27) | 0.014 | 1.05 (0.94, 1.17) | 0.392 |
|  |  |  |  |  |  |
| *Age ≥65 (n=106,318)* |  |  |  |  |  |
| PTSD | 19,916 (18.7) | 1.12 (1.07, 1.17) | <.0001 | 1.07 (1.02, 1.12) | 0.004 |
| Other Psychiatric Disorders | 25,271 (20.7) | 1.22 (1.17, 1.27) | <.0001 | 1.15 (1.10, 1.19) | <.0001 |
|  |  |  |  |  |  |
| Major Depressive Disorder | 26,909 (25.3) | 1.22 (1.17, 1.27) | <.0001 | 1.13 (1.09, 1.18) | <.0001 |
| Other Psychiatric Disorders | 18,278 (15.0) | 1.13 (1.07, 1.18) | <.0001 | 1.10 (1.05, 1.15) | <.0001 |
|  |  |  |  |  |  |
| Anxiety Disorder | 14,299 (13.4) | 1.12 (1.06, 1.18) | <.0001 | 1.06 (1.00, 1.11) | 0.037 |
| Other Psychiatric Disorders | 30,888 (25.3) | 1.21 (1.16, 1.26) | <.0001 | 1.15 (1.10, 1.19) | <.0001 |
|  |  |  |  |  |  |
| Adjustment Disorder | 7,723 (7.3) | 1.13 (1.05, 1.20) | <.0001 | 1.04 (0.98, 1.11) | 0.203 |
| Other Psychiatric Disorders | 37,464 (30.7) | 1.19 (1.15, 1.24) | <.0001 | 1.13 (1.09, 1.18) | <.0001 |
|  |  |  |  |  |  |
| Alcohol Use Disorder | 5,847 (5.5) | 1.05 (0.97, 1.15) | 0.247 | 0.99 (0.91, 1.08) | 0.802 |
| Other Psychiatric Disorders | 39,340 (32.2) | 1.20 (1.16, 1.24) | <.0001 | 1.13 (1.09, 1.17) | <.0001 |
|  |  |  |  |  |  |
| Substance Use Disorder | 3,385 (3.2) | 1.13 (1.01, 1.27) | 0.034 | 1.01 (0.90, 1.14) | 0.812 |
| Other Psychiatric Disorders | 41,802 (34.3) | 1.18 (1.14, 1.22) | <.0001 | 1.12 (1.09, 1.16) | <.0001 |
|  |  |  |  |  |  |
| Bipolar Disorder | 2,356 (2.2) | 1.41 (1.26, 1.58) | <.0001 | 1.30 (1.16, 1.46) | <.0001 |
| Other Psychiatric Disorders | 42,831 (35.1) | 1.17 (1.13, 1.21) | <.0001 | 1.11 (1.07, 1.15) | <.0001 |
|  |  |  |  |  |  |
| Psychotic Disorder | 3,552 (3.3) | 1.67 (1.54, 1.80) | <.0001 | 1.57 (1.45, 1.70) | <.0001 |
| Other Psychiatric Disorders | 41,635 (34.1) | 1.14 (1.10, 1.18) | <.0001 | 1.08 (1.04, 1.12) | <.0001 |
| RR=relative risk; CI=confidence intervals; PTSD=posttraumatic stress disorder. Reference group for each model is No Psychiatric Disorders.  Model 1: age, age squared, sex, race, and ethnicity.  Model 2: Model 1 plus obese status, diabetes, cardiovascular disease including hypertension, obstructive sleep apnea, chronic obstructive pulmonary disease, cancer, chronic kidney disease, liver disease, human immunodeficiency virus, and smoking. | | | | | |

| **Table S3.** Age-stratified (including ages <40, 40-64, ≥65) generalized linear models for associations of PTSD and other psychiatric disorders with 60-day hospitalization and death following COVID-19 infection | | | | | | | | |
| --- | --- | --- | --- | --- | --- | --- | --- | --- |
|  | **Model 1** | | | | **Model 2** | | | |
|  | *RR (95% CI)* | | *p-value* | | *RR (95% CI)* | | *p-value* | |
| **Outcome: 60-****day Hospitalizations following COVID-19 Infection** | | | | | | | | |
| *Age <40 (n=32,367; n=1,467 cases)* | |  | |  | |  | |  |
| PTSD | | 1.61 (1.40, 1.85) | | <.0001 | | 1.56 (1.35, 1.79) | | <.0001 |
| Other Psychiatric Disorders | | 1.45 (1.25, 1.68) | | <.0001 | | 1.41 (1.22, 1.64) | | <.0001 |
| No Psychiatric Disorders | | ref | | -- | | ref | | -- |
| PTSD vs. Other Psychiatric Disorders | | 1.11 (0.99, 1.24) | | 0.085 | | 1.10 (0.98, 1.23) | | 0.101 |
|  | |  | |  | |  | |  |
| *Age 40-64 (n=89,682; n=9,938 cases)* | |  | |  | |  | |  |
| PTSD | | 1.20 (1.14, 1.26) | | <.0001 | | 1.11 (1.06, 1.16) | | <.0001 |
| Other Psychiatric Disorders | | 1.27 (1.22, 1.33) | | <.0001 | | 1.15 (1.10, 1.20) | | <.0001 |
| No Psychiatric Disorders | | ref | | -- | | ref | | -- |
| PTSD vs. Other Psychiatric Disorders | | 0.94 (0.90, 0.99) | | 0.014 | | 0.97 (0.93, 1.02) | | 0.191 |
|  | |  | |  | |  | |  |
| *Age ≥65 (n=106,318; n=22,949 cases)* | |  | |  | |  | |  |
| PTSD | | 1.14 (1.11, 1.18) | | <.0001 | | 1.06 (1.02, 1.09) | | 0.001 |
| Other Psychiatric Disorders | | 1.37 (1.33, 1.40) | | <.0001 | | 1.24 (1.21, 1.28) | | <.0001 |
| No Psychiatric Disorders | | ref | | -- | | ref | | -- |
| PTSD vs. Other Psychiatric Disorders | | 0.84 (0.81, 0.86) | | <.0001 | | 0.85 (0.82, 0.88) | | <.0001 |
|  | |  | |  | |  | |  |
| **Outcome: 60-day Deaths following COVID-19 Infection** | | | | | | | | |
| *Age <40 (n=32,367; n=45 cases)* | |  | |  | |  | |  |
| PTSD | | 1.62 (0.77, 3.42) | | 0.208 | | 1.58 (0.73, 3.41) | | 0.243 |
| Other Psychiatric Disorders | | 0.76 (0.29, 1.98) | | 0.571 | | 0.73 (0.27, 1.93) | | 0.522 |
| No Psychiatric Disorders | | ref | | -- | | ref | | -- |
| PTSD vs. Other Psychiatric Disorders | | 2.13 (0.98, 4.67) | | 0.057 | | 2.17 (1.01, 4.67) | | 0.047 |
|  | |  | |  | |  | |  |
| *Age 40-64 (n=89,682; n=1,384 cases)* | |  | |  | |  | |  |
| PTSD | | 1.11 (0.97, 1.28) | | 0.128 | | 1.04 (0.91, 1.20) | | 0.562 |
| Other Psychiatric Disorders | | 1.22 (1.09, 1.38) | | 0.001 | | 1.09 (0.96, 1.23) | | 0.169 |
| No Psychiatric Disorders | | ref | | -- | | ref | | -- |
| PTSD vs. Other Psychiatric Disorders | | 0.91 (0.79, 1.05) | | 0.191 | | 0.96 (0.83, 1.10) | | 0.554 |
|  | |  | |  | |  | |  |
| *Age ≥65 (n=106,318; n=12,232 cases)* | |  | |  | |  | |  |
| PTSD | | 1.12 (1.07, 1.17) | | <.0001 | | 1.07 (1.02, 1.12) | | 0.004 |
| Other Psychiatric Disorders | | 1.22 (1.17, 1.27) | | <.0001 | | 1.15 (1.10, 1.19) | | <.0001 |
| No Psychiatric Disorders | | ref | | -- | | ref | | -- |
| PTSD vs. Other Psychiatric Disorders | | 0.92 (0.87, 0.97) | | 0.001 | | 0.93 (0.89, 0.99) | | 0.012 |
| RR=relative risk; CI=confidence intervals; PTSD=posttraumatic stress disorder.  Model 1: age, age squared, sex, race, and ethnicity.  Model 2: Model 1 plus obese status, diabetes, cardiovascular disease including hypertension, obstructive sleep apnea, chronic obstructive pulmonary disease, cancer, chronic kidney disease, liver disease, human immunodeficiency virus, and smoking. | | | | | | | | |

| **Table S4.** **Generalized linear models for associations of PTSD and adjustment disorder with 60-day hospitalization and death following COVID-19 infection (n=228,367)** | | | | | |
| --- | --- | --- | --- | --- | --- |
|  |  | **Model 1** | | **Model 2** | |
|  | *N (%)* | *RR (95% CI)* | *p-value* | *RR (95% CI)* | *p-value* |
| **Outcome: 60-day Hospitalizations following COVID-19 Infection (n=34,354 cases)** | | | | | |
| PTSD or Adjustment Disorder | 74,748 (32.7) | 1.23 (1.20, 1.26) | <.0001 | 1.13 (1.10, 1.15) | <.0001 |
| Other Psychiatric Disorders | 48,214 (21.1) | 1.32 (1.29, 1.35) | <.0001 | 1.21 (1.18, 1.24) | <.0001 |
| No Psychiatric Disorders | 105,405 (46.2) | ref | -- | ref | -- |
| PTSD/Adjustment vs. Other Psychiatric Disorders |  | 0.93 (0.91, 0.96) | <.0001 | 0.93 (0.91, 0.96) | <.0001 |
|  |  |  |  |  |  |
| **Outcome: 60-day Deaths following COVID-19 Infection (n=13,661 cases)** | | | | | |
| PTSD or Adjustment Disorder | 74,748 (32.7) | 1.14 (1.10, 1.19) | <.0001 | 1.07 (1.03, 1.12) | 0.001 |
| Other Psychiatric Disorders | 48,214 (21.1) | 1.24 (1.20, 1.29) | <.0001 | 1.17 (1.12, 1.21) | <.0001 |
| No Psychiatric Disorders | 105,405 (46.2) | ref | -- | ref | -- |
| PTSD/Adjustment vs. Other Psychiatric Disorders |  | 0.92 (0.87, 0.96) | <.0001 | 0.92 (0.88, 0.97) | 0.001 |
| RR=relative risk; CI=confidence intervals; PTSD=posttraumatic stress disorder.  Model 1: age, age squared, sex, race, and ethnicity  Model 2: Model 1 plus obese status, diabetes, cardiovascular disease including hypertension, obstructive sleep apnea, chronic obstructive pulmonary disease, cancer, chronic kidney disease, liver disease, human immunodeficiency virus, and smoking. | | | | | |

| **Table S5.** **Generalized linear models for associations of PTSD and other psychiatric disorders with 60-day hospitalization and death following COVID-19 infection, excluding breakthrough infections (n=220,449)** | | | | |
| --- | --- | --- | --- | --- |
|  | **Model 1** | | **Model 2** | |
|  | *RR (95% CI)* | *p-value* | *RR (95% CI)* | *p-value* |
| **Outcome: 60-day Hospitalizations following COVID-19 Infection** | | | | |
| PTSD | 1.18 (1.15, 1.21) | <.0001 | 1.09 (1.06, 1.12) | <.0001 |
| Other Psychiatric Disorders | 1.33 (1.30, 1.36) | <.0001 | 1.21 (1.19, 1.24) | <.0001 |
| No Psychiatric Disorders | ref | -- | ref | -- |
|  |  |  |  |  |
| **Outcome: 60-day Death following COVID-19 Infection** | | | | |
| PTSD | 1.14 (1.09, 1.19) | <.0001 | 1.08 (1.03, 1.13) | 0.001 |
| Other Psychiatric Disorders | 1.23 (1.18, 1.28) | <.0001 | 1.15 (1.10, 1.19) | <.0001 |
| No Psychiatric Disorders | ref | -- | ref | -- |
| Excluding breakthrough infections (n=7,918) defined as a positive SARS-CoV-2 test among patients with one dose of any vaccine at least 14 days prior to the test.  RR=relative risk; CI=confidence intervals; PTSD=posttraumatic stress disorder.  Model 1: age, age squared, sex, race, and ethnicity.  Model 2: Model 1 plus obese status, diabetes, cardiovascular disease including hypertension, obstructive sleep apnea, chronic obstructive pulmonary disease, cancer, chronic kidney disease, liver disease, human immunodeficiency virus, and smoking. | | | | |
